# In honour of the extraordinary career of Prof. A. James Barkovich

**DOI:** 10.4102/sajr.v24i1.2001

**Published:** 2020-11-20

**Authors:** Shalendra K. Misser, Clive Sperryn

**Affiliations:** 1 Nelson R Mandela, School of Medicine, Faculty of Radiology, University of Kwazulu-Natal, Durban, South Africa; 2 Department of Radiology, Faculty of Radiology, Lake, Smit and Partners Inc, Durban, South Africa; 3 Department of Radiology, Faculty of Radiology, Morton and Partners, Cape Town, South Africa

Professor Jim Barkovich is recognised as one of the world’s leading paediatric neuroradiologists of our time. His curriculum vitae is over 100 pages long and spans a distinguished career which began in the 1970s when he graduated with a chemistry degree (magna cum laude) and thereafter branched into medicine. He obtained his medical degree in 1980 at the George Washington University, Washington, DC. Upon completion of his radiology residency at Letterman AMC, San Francisco, in 1984, he proceeded to undertake a fellowship in neuroradiology from Walter Reed AMC, which he obtained in 1986. He has written neuroradiology textbooks and authored many book chapters, which are referred to by radiologists, the world over, in our daily practice. His book, *Paediatric Neuroradiology*, is held in exceptionally high esteem internationally. His particular contributions pertain to extensive research in brain development, neonatal brain injury, childhood epilepsy and in the last two decades the evolution of magnetic resonance imaging, especially advanced techniques such as spectroscopy. Throughout his commendable career, Prof. Barkovich has received numerous accolades, awards and recognitions from international radiological, neuroradiological and scientific societies. He has been listed as one of America’s most honoured professionals and appeared on America’s top doctors list every year since 2001. An interesting fact, less known to many, is his service on active duty in the US army from 1976 to 1989.

**FIGURE 1:**
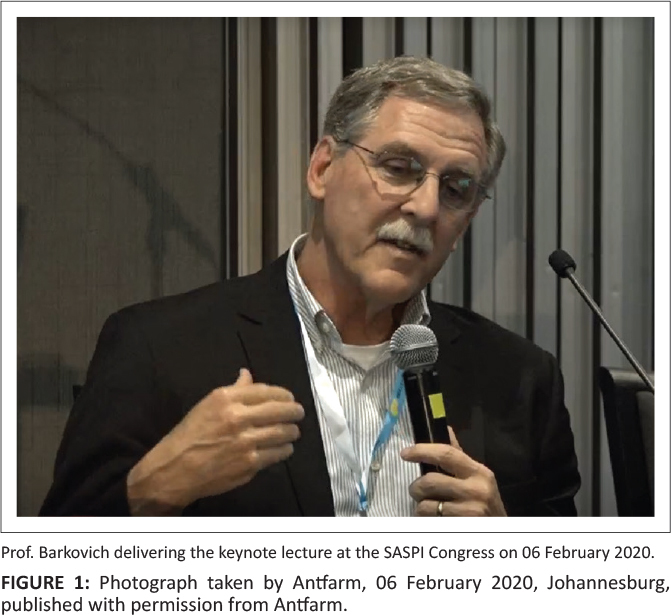
Photograph taken by Antfarm, 06 February 2020, Johannesburg, published with permission from Antfarm.

South African radiologists are fortunate that that the congress organising committee was able to persuade Prof. Barkovich to make the long journey to South Africa on two occasions (the first and third SASPI-RSSA meetings in 2012 and 2020, respectively). On both occasions he made time in his busy schedule to fly in and out of the country primarily to participate in the meetings with very little time for any personal travel or sight-seeing. His engagement with the audience is worthy of commendation and he is always happy to answer questions. The mark of greatness is the ability of a person of stature to be able to communicate equally with a training registrar as he would with a renowned fellow scientist. Prof. Barkovich was always seen chatting to attendees at our conferences irrespective of their level of training. As an incredibly modest man he serves as a role model for South African radiologists. In his laid back way he casually related to the obvious amusement of the audience, how he had used Dr Google when he came across something he had never seen before! He normalised what many of us might find ourselves doing. Apart from his excellent lectures as a keynote speaker, he contributed greatly to the stature of our meetings. Of course, many of us have been privileged to hear him speak at European and other meetings, especially through our interaction with the European Society of Neuroradiology (ESNR) over the last decade. His most recent contribution has been to act as promoter for a research project on hypoxic–ischemic injury currently being undertaken in South Africa.

**FIGURE 2:**
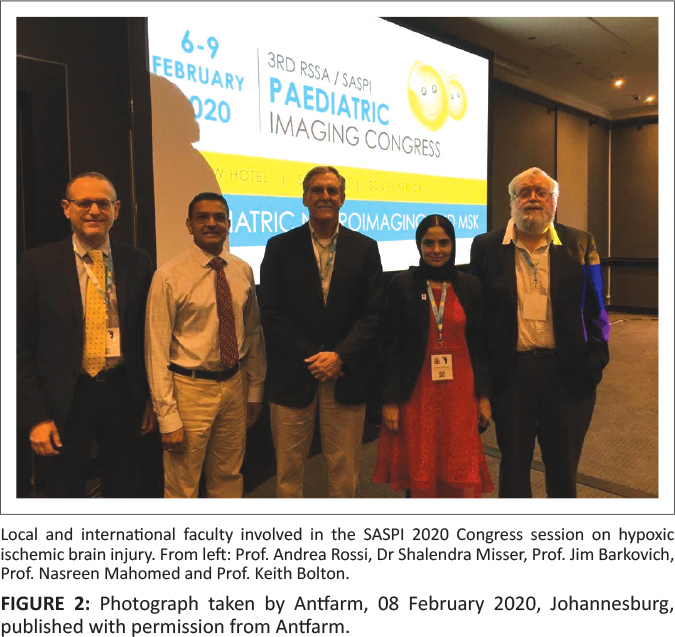
Photograph taken by Antfarm, 08 February 2020, Johannesburg, published with permission from Antfarm.

We are indeed proud of our association with Prof. Barkovich, made possible through the various societies. We take this opportunity, on behalf of all South African radiologists, to offer our congratulations in honour of his fascinating and illustrious career and we wish him well on his retirement. He is a living legend in neuroradiology circles, a humble man and a compass directing young scientists along whose path we should be walking.

